# Ischemia modified albumin can act as an independent predictor of inhospital mortality in patients with acute aortic dissection

**DOI:** 10.1038/s41598-023-27659-4

**Published:** 2023-01-07

**Authors:** Jun Xiang, Ling He, Tailuan Pen, Shuliang Wei

**Affiliations:** 1 Department of Cardiovascular Surgery, Affiliated Hospital of North Sichuan Medical College, Nanchong, 637000 Sichuan China; 2 Department of Paediatrics, Affiliated Hospital of North Sichuan Medical College, Nanchong, 637000 Sichuan China

**Keywords:** Cardiology, Risk factors

## Abstract

Acute aortic dissection (AAD) is a serious disease characterized by high mortality. However, there are no accurate indicators to predict in-hospital mortality. The objective of this study was to identify the potential value of ischemia modified albumin (IMA) in prediction of in-hospital mortality of AAD patients. This was a single-center, prospective study involved 314 patients undergoing AAD, including 197 males and 117 females, aged 26–87 (57.14 ± 21.71) years old, 116 cases of TAAD and 198 cases of TBAD (37 cases of complicated, 114 cases of high risk, and 47 cases of uncomplicated), 228 cases were underwent surgery/intervention treatment (77 cases of TAAD,151 cases of TBAD) and 86 cases were underwent conservative therapy (39 cases of TAAD, 47 cases of TBAD). The basic data, on-admission IMA level, and the all-cause in-hospital mortality was recorded. IMA in the non-survivor group and TAAD group was found to be significantly higher than that in the survivor group and TBAD group (P < 0.001). Multivariate logistic regression analysis results revealed that age (*OR* = 1.923, 95%*CI*: 1.102–4.481, *P* = 0.020), conservative therapy (*OR* = 17.892, 95%*CI*: 7.641–24.748, *P* < 0.001), D-dimer level (*OR* = 3.517, 95%*CI*: 1.874–7.667, *P* = 0.011) and IMA level (*OR* = 5.406, 95%*CI*: 2.951–10.395, *P* = 0.004) served as independent risk factors for in-hospital mortality of TAAD patients. And D-dimer level (*OR* = 2.241, 95%*CI*: 1.475–5.663, *P* = 0.018), IMA level (*OR* = 3.115, 95%*CI*: 1.792–6.925, *P* = 0.009) also served as independent risk factors for in-hospital mortality of TBAD patients, whereas surgery (*OR* = 0.110, 95%CI: 0.075–0.269, *P* < 0.001) was the protective factor of in-hospital mortality of TAAD patients. After IMA prediction, the AUC, optimal cut-off value, sensitivity, and the specificity of in-hospital mortality of AAD patients were observed to be 0.801 (95%*CI*: 0.744–0.858), 86.55 U/mL, 79.1%, and 73.2%, respectively. In addition, it was found that AUC was 0.799 (95%*CI*: 0.719–0.880) in TAAD and 0.753 (95% *CI*: 0.641–0.866) in TBAD. Overall, it was concluded that on-admission IMA level acted as an independent prediction index for in-hospital mortality of AAD patients.

## Introduction

Acute aortic dissection (AAD) is a serious type of acute aortic syndrome encountered in clinical practice and is also regarded as one of the most severe diseases in cardiovascular surgery with acute onset as well as high mortality. If not intervened in time, the mortality can effectively increase by 1–2% per hour within 48 h and reach approximately 90% in 1 week^1,2^. Therefore, rapid diagnosis and timely treatment are of great significance to mitigate mortality. Surgery is an effective way to save the lives of patients, but there are no accurate indicators to predict the potential effect of treatment. The traditional diagnosis of AAD primarily relies on the clinical symptoms and computed tomography angiography (CTA), but in recent years and it has been reported that various biological markers can exhibit significant potential for the rapid diagnosis of AAD^3–6^.

Ischemia modified albumin (IMA) is a recently discovered biochemical marker for acute myocardial ischemia in recent years. It was first discovered in 1990 and increase in its level may be related to the marked decrease in the binding ability of free metal ions (e.g., Ni2^+^, Cu2^+^, Co2^+^) caused by amino N-terminal sequence changes of albumin during the process of ischemia. The protein that was produced by this chemical reaction is called IMA, and it generally disappears within a few minutes after the myocardial ischemia and hypoxia. As a result, it can be effectively used as an important basis for facilitating early diagnosis and risk stratification biomarker for the acute coronary syndrome^7^. It has also been approved by the Food and Drug Administration (FDA) of U.S. for the auxiliary diagnosis of early myocardial ischemia^8^. Many prior studies have demonstrated that the IMA level can also increase in non-cardiac diseases (e.g., pulmonary embolism, diabetes, severe sepsis, intestinal ischemia), and it has been closely linked to the prognosis^9–12^. However, only few reports describing the possible applications of IMA in AAD have been published previously and the value of prognosis have been reported. For instance, Eroglu et al.^4^ demonstrated that the IMA level of AAD patients in the serum was significantly higher than that of the healthy people, thus suggesting that IMA level could be potentially related to the occurrence of AAD. However, whether IMA can exhibit predictive value for the prognosis of AAD patients is not completely clear. Hence, the current study aimed to explore the value of IMA in prediction of in-hospital mortality of AAD patients by detecting on-admission IMA levels and thus providing an effective prognostic factor for the prognosis of AAD.

## Methods

### Patient enrollment

The clinical data of various AAD patients hospitalized in the Department of Cardiovascular Surgery in the Affiliated Hospital of North Sichuan Medical College from July 2016 to December 2020 due to the clinical manifestation of acute chest and back pain were collected. All the patients were diagnosed with AAD by thoracic-abdominal aortic tomography angiography. All the subtype dissection were defined by Society for Vascular Surgery (SVS) and Society of Thoracic Surgeons (STS)^13^. An aortic dissection with an entry tear in ascending aorta was classified as Stanford type A aortic dissection (TAAD) whereas an aortic dissection with an entry tear in descending aorta or beyond was classified as Stanford type B aortic dissection (TBAD). In addition, according to the comorbidities and risk factors, TBAD was classified as complicated dissection (rupture, malperfusion), high risk dissection (refractory pain, refractory hypertension, bloody pleural effusion, aortic diameter > 40 mm, radiographic only malperfusion, readmission, entry tear: lesser curve location, false lumen diameter > 22 mm), uncomplicated dissection (lack of rupture, malperfusion and high-risk features).

Inclusion criteria used were: (1) AAD confirmed by CTA of the thoracic-abdominal aorta (both TAAD and TBAD); (2) age > 18 years; (3) time from onset to the medical consultation < 24 h; (4) Completion of informed consent form.

Exclusion criteria used were: (1) history of malignant tumor; (2) acute myocardial infarction(both ECG characteristic changes (ST segment elevation, pathological Q wave, etc.) and myocardial zymogram elevation), pulmonary embolism, and sepsis; (3) pregnant women; (4) patients with incomplete or missing clinical data.

All the patients were admitted to the intensive care unit and were given sedation, analgesia, as well as oxygen inhalation and they maintained a normal bowel movement. Urapidil and sodium nitroprusside were used to control the blood pressure within 120/80 mmHg (1 mmHg = 0.133 kPa), and β-receptor blockers were used to control the heart rate lower than 80/min. For patients with TAAD, emergency surgery was performed unless the family refused to give the consent. The patients with complicated and high risk TBAD were treated with thoracic endovascular aortic repair (TEVAR). Moreover, uncomplicated TBAD wear treated with optimal medical therapy (OMT)^13,14^. The study was conducted in accordance with the Declaration of Helsinki (as revised in 2013).This study was approved by the Medical Ethics Committee of the Affiliated Hospital of North Sichuan Medical College (No. 2018 ERA037) and informed consent was obtained from all the patients.

### Detection of IMA level

Five milliliters of venous blood were extracted from the peripheral vein of all AAD patients within two hours after admission. After incubation for 20 min, the blood samples were centrifuged at 3500 rpm for 10 min and the serum was collected. The albumin cobalt-binding (ACB) test was used to detect IMA levels (IMA reagents were purchased from Yikang Science Technique Development Co., Changsha, China). All operations were conducted in strict accordance with the instructions provided by the manufacturer.

### Indicators

Based on the observed mortality in the patients during hospitalization, they were divided into survivor and non-survivor groups. The preoperative variables of the patients in the two groups were recorded including the various basic information (e.g., age, sex, body mass index, weight, blood pressure, family history, diabetes history, cerebral infarction history, smoking history, drinking history, and dissection type). In addition, the preoperative laboratory examinations (e.g., white blood cell count, red blood cell count, platelet count, hemoglobin, IMA, D-dimer, C-reactive protein, alanine aminotransferase, aspartame acid aminotransferase, albumin, bilirubin, creatinine, Cardiac troponin T, and creatine kinase isoenzyme, and the different treatment methods used (e.g., conservative therapy, open surgery, and TEVAR) were also noted. The endpoint of this study was in-hospital mortality. The relevant variables of the patients in the two groups were statistically compared and the different risk factors were screened.

### Statistical analysis

SPSS 20.0 software was used for the statistical analysis and the results have been expressed as $\overline{x}$ ± *s*. and the comparison between the two groups was carried out by Student’s t-test. The categorical variables were expressed by frequency (rate or percentage), and comparison between the two groups was by chi-square test. The variables with statistical significance after the Univariate analysis were further validated with multivariate logistic regression analysis to explore the risk factor of in-hospital mortality of AAD patients. The receiver operating characteristic (ROC) curve was used for determining the diagnostic efficacy. The area under curve (AUC), sensitivity, and specificity were calculated respectively to determine the value of IMA in the prediction of in-hospital mortality of AAD patients. It was found out that all the values of P were two-sided, and a P < 0.05 was considered as statistically significant.

## Results

### Basic information of AAD patients

**Figure 1:**
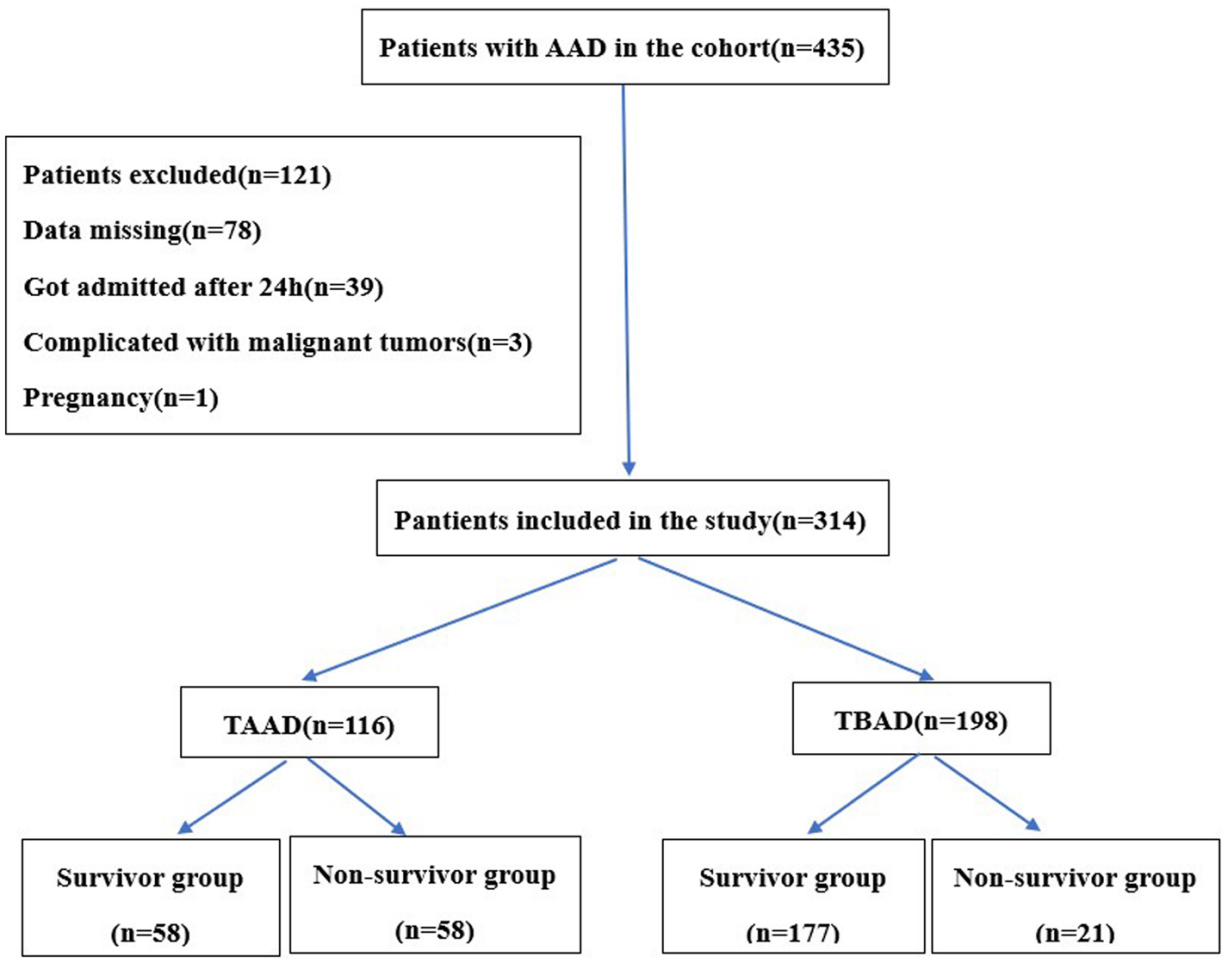
Flow chart depicting patient enrollment and the study design. AAD, acute aortic dissection; TAAD, Stanford type A aortic dissection; TBAD, Stanford type B aortic dissection.

A total of 435 cases of AAD patients were enrolled in this study. After screening according to exclusion criteria, 314 cases of patients with AAD were enrolled (Fig. 1). These included 197 males and 117 females, aged 26–87 (57.14 ± 21.71) years old, 116 cases of TAAD and 198 cases of TBAD (37 cases of complicated, 114 cases of high risk, and 47 cases of uncomplicated), 228 cases of surgery/intervention treatment (77 cases of TAAD, 151 cases of TBAD) and 86 cases of conservative therapy (39 cases of TAAD, 47 cases of TBAD and the main reasons for selection of conservative therapy by the TAAD patients were old age and fear of high costs). Moreover, 235 survivors and 79 non-survivors during the hospitalization were found with the mortality rate of 25.2%, among which 53 cases died under conservative therapy (39 cases of TAAD, 14 cases of TBAD) and the cause of death was dissection rupture. In addition, 19 cases died from major complications from surgery, including 9 cases of postoperative lung complication, 5 cases of renal insufficiency, 2 cases of cerebral infarction, 2 cases of cardiac insufficiency, and1 case of gastrointestinal hemorrhage. 7 cases died after TEVAR with the causes including 3 cases of pulmonary complication, 2 cases of cerebral infarction, 1 case of renal insufficiency, and 1 case of exception of the dissection at 10 days after the first operation. This patient was recommended to undergo the second operation due to the large distal rupture and increase in the false lumen after the first operation but refused (Table 1).

**Table 1:** General characteristics of AAD patients.

Number of patients	314
Age (year)	57.14 (26–87)
**Sex (n, %)**	
Male	197 (62.7%)
Female	117 (37.3%)
**TAAD (n, %)**	116 (36.9%)
Conservative therapy	39 (33.6%)
Surgical	77 (66.4%)
**TBAD (n, %)**	198 (63.1%)
Complicated	37 (18.7%)
High risk	114 (57.6%)
Uncomplicated	47 (23.7%)
Conservative therapy	47 (23.7%)
TEVAR	151 (76.3%)
**The cause of death (n, %)**	
Aortic dissection rupture	54 (68.4%)
Pulmonary complications	12 (15.2%)
Renal failure	6 (7.6%)
Cerebral infarction	4 (6.1%)
Heart failure	2 (2.5%)
Gastrointestinal bleeding	1 (1.3%)

AAD, acute aortic dissection; TAAD,Stanford type A aortic dissection; TBAD, Stanford type B aortic dissection; TEVAR, thoracic endovascular aortic repair.

### Related risk factors for in-hospital mortality in patients with TAAD and TBAD

**Figure 2:**
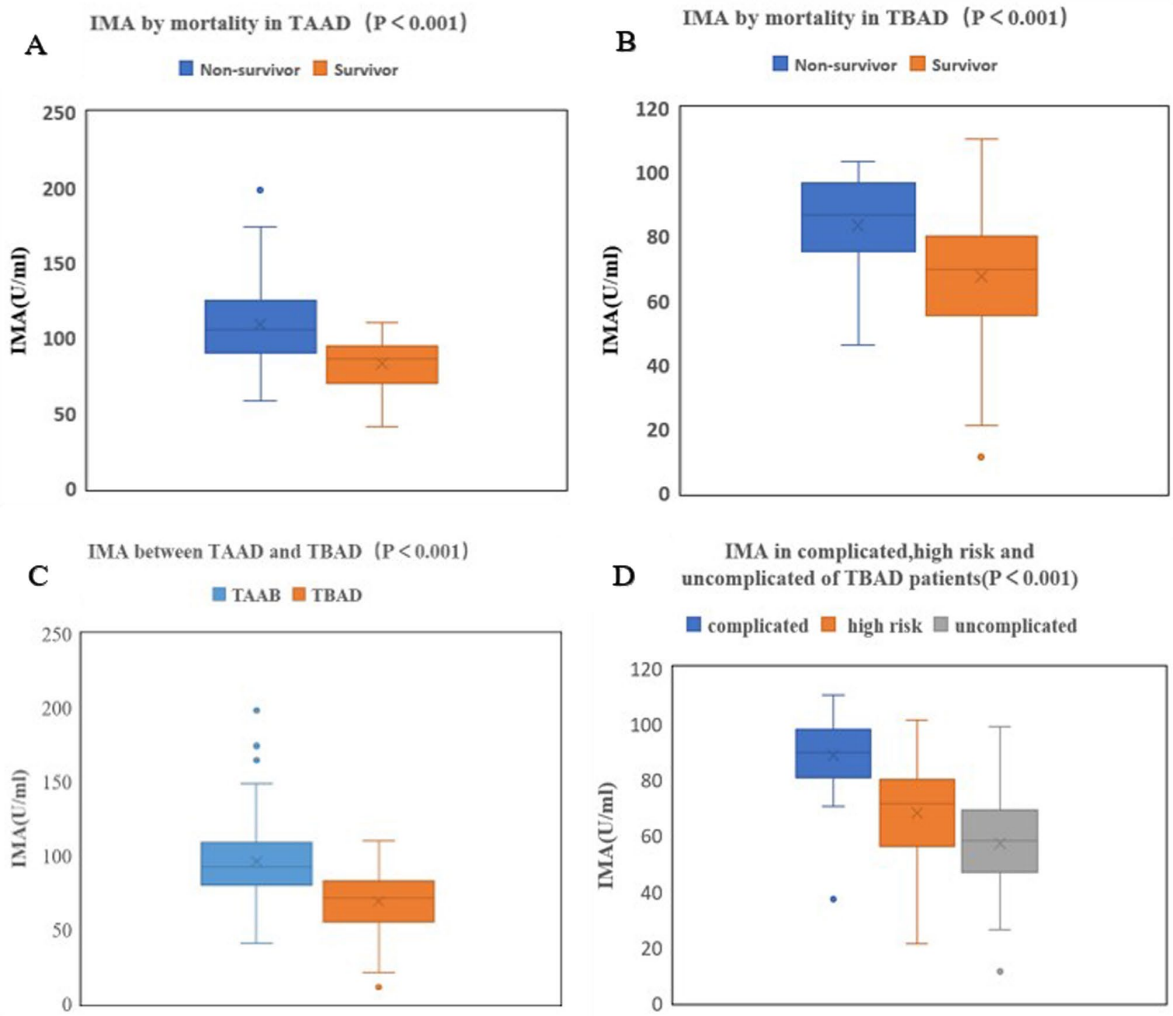
Comparison of IMA between different groups. (**A**) Shows IMA between non-survivor group and survivor group in TAAD. (**B**) Shows IMA between non-survivor group and survivor group in TBAD. (**C**) Shows IMA between TAAD and TBAD. (**D**) Shows IMA between complicated dissection, high risk dissection and uncomplicated dissection. IMA, Ischemia-modified albumin; TAAD, Stanford type A aortic dissection; TBAD, Stanford type B aortic dissection. The bottom and top edges of each box represent the first and third quartiles, respectively, the band within the box represents the median value.

It was found that compared with the survivor group in patients with TAAD, the patients in the non-survivor group were older, showed lower platelet count, higher white blood cell count, IMA (Fig. 2A), D-dimer, C-reactive protein, creatinine, and cTnT, and a higher proportion of pericardial effusion and conservative treatment (*P* < 0.05, Table 2). And compared with the survivor group in patients with TBAD, the patients in the non-survivor group were also older, showed higher IMA (Fig. 2B), higher D-dimer, and a higher proportion of conservative treatment (*P* < 0.05, Table 3). IMA in TAAD group was found to be markedly higher than that in the TBAD group (96.22 ± 25.45 vs. 69.25 ± 19.07, *P* < 0.001, Fig. 2C). The IMA in non-survivor group was much higher than that in the survivor group in both TAAD (109.15 ± 26.91 vs. 83.29 ± 15.58, *P* < 0.001) and TBAD (83.50 ± 15.66 vs. 67.56 ± 18.76, *P* < 0.001) patients (Table 4), and the IMA in complicated TBAD patients was substantially higher than that in high risk and uncomplicated TBAD patients (88.57 ± 13.48 vs. 68.24 ± 16.78 vs. 57.16 ± 16.55, *P* < 0.001, Fig. 2D). The results of multivariate logistic regression analysis revealed that age (*OR* = 1.923, 95%*CI*: 1.102 ~ 4.481, *P* = 0.020), conservative therapy (*OR* = 17.892, 95%*CI*: 7.641–24.748, *P* < 0.001), D-dimer level (*OR* = 3.517, 95%*CI*: 1.874–7.667, *P* = 0.011) and IMA level (*OR* = 5.406, 95%*CI*: 2.951 ~ 10.395, *P* = 0.004) served as independent risk factors for in-hospital mortality of TAAD patients (Table 5). And D-dimer level (*OR* = 2.241, 95%*CI*: 1.475–5.663, *P* = 0.018), IMA level (*OR* = 3.115, 95%*CI*: 1.792–6.925, *P* = 0.009) also served as independent risk factors for in-hospital mortality of TBAD patients (Table 6). However, surgery (*OR* = 0.110, 95%CI: 0.075–0.269, *P* < 0.001) could be considered as potential protective factors for in-hospital mortality of TAAD patients.

**Table 2:** Univariate analyses of variables associated with in-hospital mortality in TAAD.

Variable	Survivor (*n* = 58)	Non-survivor (*n* = 58)	*t*/χ^2^	*P value*
Age (years)	54.39 ± 10.37	60.93 ± 11.77	− 3.175	0.002
BMI (kg/m^2^)	24.38 ± 4.27	24.65 ± 4.43	− 0.334	0.738
SBP (mmHg)	156.45 ± 41.45	141.62 ± 40.17	1.956	0.053
DBP (mmHg)	94.26 ± 20.32	91.35 ± 21.14	0.755	0.451
WBC (× 10^9^/L)	11.21 ± 3.04	12.55 ± 3.37	− 2.248	0.026
RBC (× 10^12^/L)	4.41 ± 1.17	4.19 ± 1.23	0.987	0.326
PLT (× 10^9^/L)	175.47 ± 43.29	155.33 ± 51.58	2.277	0.025
Hb (g/L)	127.51 ± 23.25	123.66 ± 23.38	0.889	0.375
IMA (U/mL)	83.29 ± 15.58	109.15 ± 26.91	− 6.334	< 0.001
D-dimer (mg/L)	3.21 ± 2.35	5.54 ± 3.08	− 4.580	< 0.001
CRP (mg/L)	11.76 ± 5.67	13.97 ± 5.95	− 2.047	0.043
ALT (U/L)	32.18 ± 13.76	37.01 ± 12.52	− 1.977	0.051
AST (U/L)	30.84 ± 12.15	34.65 ± 11.75	− 1.716	0.088
ALB (g/L)	38.65 ± 5.44	37.76 ± 5.05	0.363	0.913
TB (μmol/L)	15.52 ± 4.67	17.04 ± 4.78	− 1.732	0.086
Cr (μmol/L)	84.27 ± 27.64	95.32 ± 25.95	− 2.219	0.028
cTnT (pg/ml)	9.03 ± 3.47	11.64 ± 4.79	− 2.073	0.040
CK-MB (ng/ml)	2.83 ± 0.89	3.01 ± 0.97	− 1.041	0.299
**Gender**			0.149	0.699
Male	38 (65.52%)	36 (62.07%)		
Female	20 (34.48%)	22 (37.93%)		
**Smoking**			0.037	0.848
Yes	36 (62.07%)	37 (63.79%)		
No	22 (37.93%)	21 (36.21%)		
**Drinking**			0.310	0.577
Yes	28 (48.27%)	31 (53.45%)		
No	30 (51.73%)	27 (46.55%)		
**Hypertension**			0.904	0.342
Yes	51 (87.93%)	54 (93.10%)		
No	7 (13.07%)	4 (6.90%)		
**Cerebral infarction**			0.537	0.464
Yes	3 (5.17%)	5 (8.62%)		
No	55 (94.83%)	53 (91.38%)		
**Diabetes**			0.325	0.569
Yes	6 (10.34%)	8 (13.79%)		
No	52 (89.66%)	50 (86.21%)		
**Pericardial effusion**			4.802	0.028
Yes	34 (58.62%)	45 (77.59%)		
No	24 (41.38%)	13 (22.41%)		
**Management**			94.532	< 0.001
Conservative therapy	0 (0%)	39 (67.24%)		
SURGICAL	58 (100.00%)	19 (32.76%)		

BMI, body mass index; SBP, systolic blood pressure; DBP, diastole blood pressure; WBC, white blood cell; RBC, red blood cell; PLT, platelets; Hb, hemoglobin; IMA, ischemic modified albumin; CRP, C-reactive protein; ALT, alanine transaminase; AST, aspartate aminotransferase; ALB, albumin; TB, total bilirubin; Cr, creatinine; cTnT, troponin T; CK-MB, creatine kinase MB; TAAD ,Stanford type A aortic dissection.

**Table 3:** Univariate analyses of variables associated with in-hospital mortality in TBAD.

Variable	Survivor (*n* = 177)	Non-survivor (*n* = 21)	*t*/χ^2^	*P value*
Age (years)	55.02 ± 9.74	59.54 ± 11.08	− 1.566	0.049
BMI (kg/m^2^)	24.62 ± 3.87	24.94 ± 4.01	− 0.356	0.721
SBP (mmHg)	151.47 ± 38.79	148.61 ± 41.29	0.317	0.751
DBP (mmHg)	95.78 ± 21.18	87.69 ± 20.44	1.660	0.098
WBC (× 10^9^/L)	11.53 ± 3.24	12.33 ± 3.07	− 1.075	0.283
RBC (× 10^12^/L)	4.33 ± 1.20	4.31 ± 1.19	0.072	0.942
PLT (× 10^9^/L)	198.35 ± 44.92	190.67 ± 47.38	0.736	0.462
Hb (g/L)	131.66 ± 24.17	128.65 ± 24.76	0.538	0.591
IMA (U/mL)	67.56 ± 18.76	83.50 ± 15.66	− 3.793	< 0.001
D-dimer (mg/L)	3.41 ± 2.08	5.38 ± 2.95	− 3.906	< 0.001
CRP (mg/L)	10.95 ± 4.98	11.45 ± 4.63	− 0.438	0.661
ALT (U/L)	36.61 ± 12.71	35.54 ± 12.82	0.364	0.715
AST (U/L)	32.55 ± 12.74	29.23 ± 11.87	1.136	0.257
ALB (g/L)	38.88 ± 5.64	39.45 ± 5.21	− 0.441	0.659
TB (μmol/L)	15.76 ± 4.54	15.98 ± 4.21	− 0.211	0.832
Cr (μmol/L)	86.15 ± 25.33	95.68 ± 24.65	− 1.634	0.103
cTnT (pg/mL)	8.87 ± 3.31	8.66 ± 3.19	0.275	0.782
CK-MB (ng/mL)	2.87 ± 0.91	3.11 ± 1.04	− 1.125	0.261
**Gender**			0.947	0.330
Male	112 (63.28%)	11 (52.38%)		
Female	65 (36.72%)	10 (47.62%)		
**Smoking**			0.001	0.983
Yes	110 (62.15%)	13 (61.91%)		
No	67 (37.85%)	8 (38.09%)		
**Drinking**			1.073	0.300
Yes	80 (45.20%)	7 (33.33%)		
No	97 (54.80%)	14 (66.67%)		
**Hypertension**			0.205	0.651
Yes	150 (84.75%)	17 (80.95%)		
No	27 (15.25%)	4 (19.05%)		
**Cerebral infarction**			2.419	0.120
Yes	15 (8.48%)	4 (19.05%)		
No	162 (92.52%)	17 (80.95%)		
**Diabetes**			0.060	0.807
Yes	20 (11.30%)	2 (9.52%)		
No	157 (88.70%)	19 (90.48%)		
**Pleural effusion**			0.500	0.579
Yes	31 (17.51%)	5 (23.81%)		
No	146 (82.49%)	16 (76.19%)		
**Management**			13.915	0.001
Conservative therapy	33 (18.64%)	14 (66.67%)		
TEVAR	144 (81.36%)	7 (33.33%)		

BMI, body mass index; SBP, systolic blood pressure; DBP, diastole blood pressure; WBC, white blood cell; RBC, red blood cell; PLT, platelets; Hb, hemoglobin; IMA, ischemic modified albumin; CRP, C-reactive protein; ALT, alanine transaminase; AST, aspartate aminotransferase; ALB, albumin; TB, total bilirubin; Cr, creatinine; cTnT, troponin T; CK-MB, creatine kinase MB; TBAD, Stanford type B aortic dissection; TEVAR, thoracic endovascular aortic repair.

**Table 4:** IMA in non-survivor and survivor groups between TAAD and TBAD patients.

Type of AAD	Non-survivor group	Survivor group	χ^2^	P value
TAAD (n = 116)	109.15 ± 26.91	83.29 ± 15.58	6.334	< 0.001
TBAD (n = 198)	83.50 ± 15.66	67.56 ± 18.76	3.739	< 0.001

AAD, acute aortic dissection; TAAD, Stanford type A aortic dissection; TBAD Stanford type B aortic dissection; IMA, ischemic modified albumin.

**Table 5:** Multivariate logistic regression analysis of the different variables associated with in-hospital mortality in TAAD.

Clinical variables	OR	95% CI	P value
Age	1.923	1.102–4.481	0.020
IMA	5.406	2.951–10.395	0.004
D-dimer	3.517	1.874–7.667	0.011
Conservative therapy	17.892	7.641–24.748	< 0.001
Surgical	0.110	0.075–0.269	< 0.001

TAAD, Stanford type A aortic dissection; IMA, ischemic modified albumin; OR, odds ratio; CI, confidence interval.

**Table 6:** Multivariate logistic regression analysis of the different variables associated with in-hospital mortality in TBAD.

Clinical variables	OR	95% CI	P value
IMA	3.115	1.792–6.925	0.009
D-dimer	2.241	1.475–5.663	0.018

TBAD, Stanford type B aortic dissection; IMA, ischemic modified albumin; OR, odds ratio; CI, confidence interval.

### Subgroup analyses

Subgroup analysis was conducted to determine the possible correlation between IMA and in-hospital mortality across comorbidities and different parameters and the results have been shown in Table 5. It was found that the stratification factors did not have a significant impact on the relationship between IMA and in-hospital mortality (interaction P-value > 0.05). Besides, the results of the study indicated that in all the subgroups, the increase in IMA levels was closely related to the increase in the in-hospital mortality of AAD patients (Table 7).

**Table 7:** Subgroup analysis of the correlation between IMA and in-hospital mortality of patients with AAD.

Subject		N	IMA		*P* for interaction
			Low (< 86.55)	High (≥ 86.55)	
TAAD					0.203
	Yes	116	1.0 (ref.)	5.177 (3.733–11.130)	
	No	198	1.0 (ref.)	2.358 (1.153–3.218)	
Age					0.406
	≤ 60 year	161	1.0 (ref.)	3.784 (1.547–4.940)	
	> 60 year	153	1.0 (ref.)	3.847 (1.593–5.383)	
RI					0.371
	Yes	30	1.0 (ref.)	6.652 (5.547–14.758)	
	No	284	1.0 (ref.)	4.121 (2.976–9.983)	

AAD, acute aortic dissection; TAAD, Stanford type A aortic dissection; IMA, ischemic modified albumin; RI, renal insufficiency.

### Sensitivity and specificity of IMA in predicting in-hospital mortality

**Figure 3:**
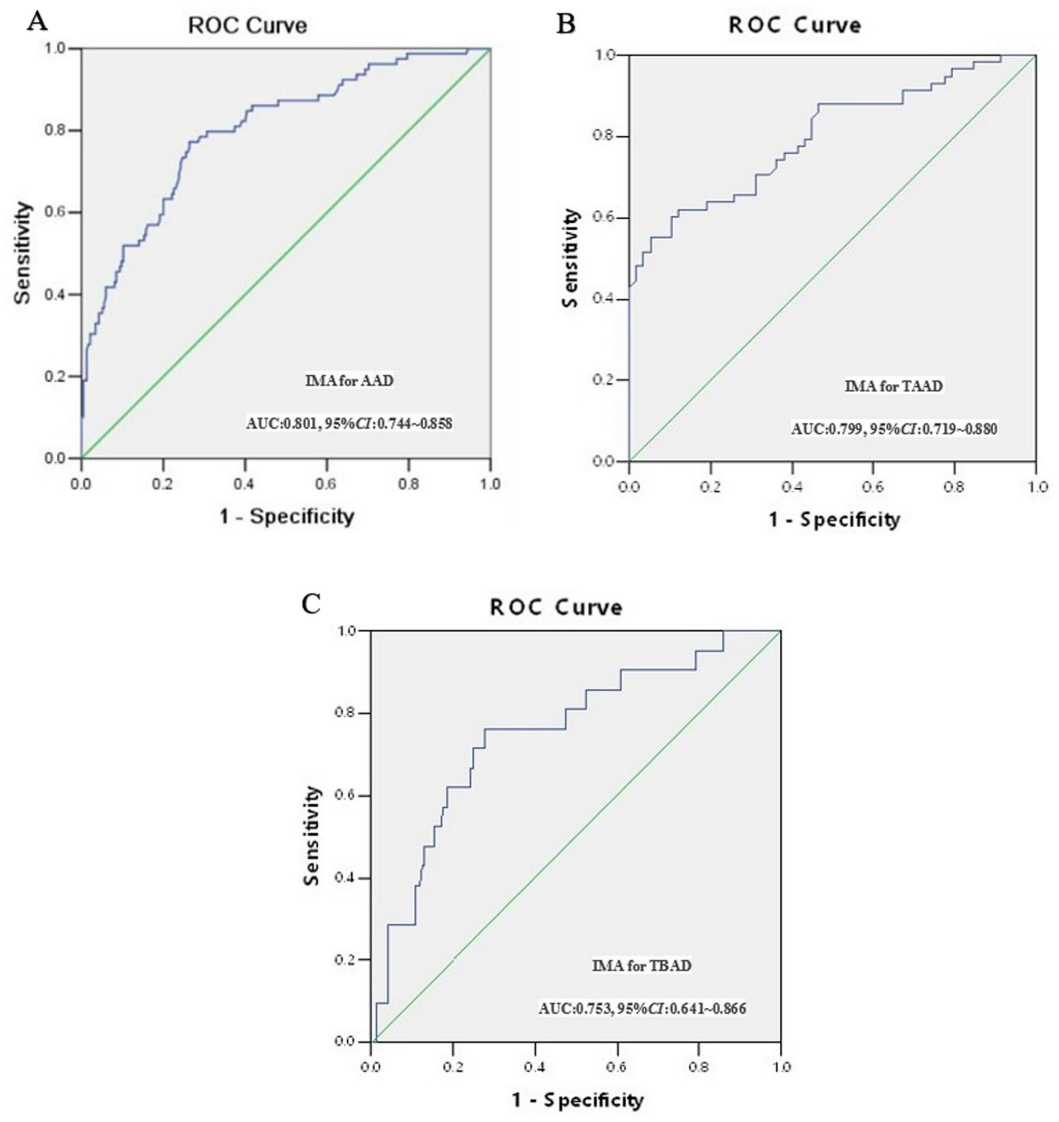
AUC value of IMA for predicting in-hospital mortality in patients with different types of AAD. IMA, Ischemia-modified albumin; AAD, acute aortic dissection; TAAD, Stanford type A aortic dissection; ROC, Receiver operating characteristic; AUC, area under the curve.

ROC analysis was performed to determine the possible cut-off value of IMA in predicting in-hospital mortality. It was observed that IMA can be used as an important indicator for predicting in-hospital mortality of AAD patients with AUC of 0.801 (95% *CI*: 0.744–.858), the optimal cut-off value of 86.55 U/mL, the sensitivity of 79.1%, and the specificity of 73.2% (Fig. 3A). Interestingly, IMA can also predict the in-hospital mortality of both TAAD (Fig. 3B) and TBAD (Fig. 3C) patients well with the AUC of 0.799 (95% *CI*: 0.719–0.880) and 0.753 (95% *CI*: 0.641–0.866), respectively (Table 8).

**Table 8:** Diagnostic value of IMA for predicting in-hospital mortality.

	AUC	Cut-off value	95% CI	Sensitivity (%)	Specificity (%)
AAD	0.801	86.55	0.744–0.858	79.1	73.2
TAAD	0.799	99.26	0.719–0.880	72.1	87.9
TBAD	0.753	79.38	0.641–0.866	76.2	72.3

IMA, ischemic modified albumin; AUC, area under the curve; CI, confidence interval, AAD, acute aortic dissection; TAAD, Stanford type A aortic dissection; TBAD, Stanford type B aortic dissection.

## Discussion

In this article, the value of IMA in predicting in-hospital mortality of 314 patients who were subjected to AAD were assessed. The main findings of this study were that preoperative IMA was significantly associated with an increased risk of in-hospital mortality in patients with AAD (both TAAD and TBAD), and IMA in TAAD was found to be significantly higher than that in the TBAD. Thus, IMA can function as an independent predictor of in-hospital mortality in patients with AAD.

AAD is one of the most common emergencies in cardiovascular surgery which can display symptoms of rapid onset, rapid progress, and high mortality^15,16^. Some prior reports have demonstrated that age, pleural effusion, Marfan syndrome, and creatinine are important risk factors linked with increased death of AAD patients^16–18^. The results of this study revealed that age, TAAD, IMA, D-dimer, as well as conservative therapy served as independent risk factors for in-hospital mortality of AAD patients, and surgery/intervention acted as a protective factor for AAD. This was consistent with the findings of both An et al.^19^ and Uchida et al.^20^.

IMA is a new biochemical marker for acute myocardial ischemia and can as an important biomarker for the early diagnosis and risk stratification in acute coronary syndrome. For example, Bhakthavatsala et al*. *^21^ reported that the sensitivity of IMA for the diagnosis of the acute coronary syndrome was 92%, specificity was 87%, and the IMA level was found to be positively related to the severity of the lesion. In addition, some authors have reported that IMA was found to be a more sensitive indicator than TnI, myoglobin, and CK-MB in acute coronary syndrome, because IMA levels can rise within 30 min and then continue to increase for the next 6–12 h. Eroglu et al*. *^4^ demonstrated that the IMA level of AAD patients in the serum was significantly higher than that of the healthy people, thus suggesting that IMA level may be related to the occurrence of AAD. The IMA level in serum of AAD patients was significantly increased, and the increase in TAAD was observed to be more obvious^22^. However, Sbarouni et al.^23^ found that on-admission IMA levels of AAD patients were not significantly increased as compared to the control group and did not increase even after the surgery. These results, which w contrasting to our major findings may be attributed to the fact that reported data by this group included fewer cases (46 cases) compared to our study. In addition, the control group included patients with chronic aortic aneurysms, without risk of coronary heart disease, and patients with sepsis, occult coronary heart disease, and diabetes were not excluded, thereby causing a higher IMA baseline level in the control group. It is worth indicating that optimal IMA test time was also missed because the doctor’s office visit was 23 ± 17 h after the onset of symptoms. Our study showed that, IMA in TAAD group was substantially higher as compared to the TBAD group. This may be related to myocardial ischemia or malperfusion caused by TAAD. Our study also found that upon comparison of the different subtype of TBAD, IMA in complicated patients was markedly greater than that in high risk and uncomplicated patients, and this has not been reported in previous studies.

To date, there are only a few studies elaborating the value of IMA in prognosis prediction, especially on AAD prognosis. Yang et al.^22^ investigated the IMA levels of 731 AAD patients and found that IMA was an independent prediction factor of poor prognosis for AAD patients. In addition, the optimal cut-off value of prognosis was 79.35 U/mL, whereas the specificity and sensitivity were 84.8% and 80.6%, respectively. This conclusion might have similar significance in sepsis, acute coronary syndrome, and other diseases. Yin et al.^24^ investigated 117 sepsis cases and found that augmented IMA level ≥ 110 U/mL could significantly increase the mortality of the sepsis patients, which could serve as an important predictive factor of in-hospital mortality. Consuegra et al.^25^ also suggested that IMA could act as a predictive factor for long-term adverse outcomes in patients with acute chest pain. The results of this study revealed that the IMA level of the non-survivor group was significantly higher than that of the survivor group and IMA can function as an independent predictor of in-hospital mortality in AAD patients with the AUC of 0.801 (95% *CI*: 0.744–0.858). It could be due to the non-survivor group displaying a higher proportion of TAAD, a wider range of damage and more severe ischemia. The results of multivariate logistic regression analysis also indicated that IMA was an independent influencing factor of in-hospital mortality for both TAAD and TBAD patients, and the predicted AUC of in-hospital mortality were 0.799 and 0.753, the optimal cut-off value were 99.26 U/mL and 79.38 U/mL, the sensitivity was 72.1% and 76.2%, and the specificity was 87.9% and 72.3%, respectively. Our study distinguished the predictive value of IMA for different types of AAD and compared the IMA levels in different subtypes of TBAD while study published by Yang et al*.*^22^ did not explore these parameters. Overall, these findings indicated that high IMA level can lead to increased mortality of AAD patients and IMA could be potentially used as a prediction index for poor prognosis and in-hospital mortality of AAD patients.

A number of previous studies have also found that D-dimer is a predictor of in-hospital mortality in patients with AAD^3,6^. However, D-dimer is a non-specific parameter, as it functions as a specific fibrinolytic marker, displaying specific diagnosis and prediction values for the diseases with thrombotic tendency (e.g., pulmonary embolism, deep vein thrombosis). It has been reported to increase 2 h after thrombosis and therefore has little clinical application^26^. IMA appears within minutes of ischemia or hypoxia, detected easily, have a high sensitivity as well as specificity, and thus it might be a relatively better biomarker than D-dimer in predicting the in-hospital mortality in patients with AAD.

However, our study has few limitations. First, the sample size of this study was limited, lacked multi-center large samples, and the long-term risks and benefits of discharged patients were not followed up. Second, we did not separate patients for conducting statistical analysis to determine the predictive value of in-hospital mortality after surgery or intervention. Third, we did not analyze the medium and long-term outcomes of patients with different IMA levels. Therefore, more rigorous multicenter prospective randomized controlled studies are needed in future to further corroborate our findings.

## Conclusions

The main advantage of using IMA as a potential biomarker in comparison to other available markers is its ability to detect ischemic conditions at earlier stages^27^. As a rapid detection index, IMA was found to be a simple measurement method, and blood can be drawn for testing upon admission or in the emergency department. In addition, it can be used for the simple risk assessment of AAD patients. Overall, on-admission IMA level served as an independent prediction index of in-hospital mortality for AAD patients with a significantly higher prediction value.

## Data Availability

The datasets generated and analyzed during the current study could be obtained from the corresponding author upon reasonable request.
